# Cryptic-Prophage-Encoded Small Protein DicB Protects *Escherichia coli* from Phage Infection by Inhibiting Inner Membrane Receptor Proteins

**DOI:** 10.1128/JB.00475-19

**Published:** 2019-11-05

**Authors:** Preethi T. Ragunathan, Carin K. Vanderpool

**Affiliations:** a Department of Microbiology, University of Illinois at Urbana-Champaign, Urbana, Illinois, USA; b Carl R. Woese Institute for Genomic Biology, Urbana, Illinois, USA; Geisel School of Medicine at Dartmouth

**Keywords:** Hfq, cell division, prophages, small RNA, small protein, sugars

## Abstract

Bacterial genomes harbor cryptic prophages that have lost genes required for induction, excision from host chromosomes, or production of phage progeny. Escherichia coli K-12 strains contain a cryptic prophage, Qin, that encodes a small RNA, DicF, and a small protein, DicB, that have been implicated in control of bacterial metabolism and cell division. Since DicB and DicF are encoded in the Qin immunity region, we tested whether these gene products could protect the E. coli host from bacteriophage infection. Transient expression of the *dicBF* operon yielded cells that were ∼100-fold more resistant to infection by λ phage than control cells, and the phenotype was DicB dependent. DicB specifically inhibited infection by λ and other phages that use ManYZ membrane proteins for cytoplasmic entry of phage DNA. In addition to blocking ManYZ-dependent phage infection, DicB also inhibited the canonical sugar transport activity of ManYZ. Previous studies demonstrated that DicB interacts with MinC, an FtsZ polymerization inhibitor, causing MinC localization to midcell and preventing Z ring formation and cell division. In strains producing mutant MinC proteins that do not interact with DicB, both DicB-dependent phenotypes involving ManYZ were lost. These results suggest that DicB is a pleiotropic regulator of bacterial physiology and cell division and that these effects are mediated by a key molecular interaction with the cell division protein MinC.

**IMPORTANCE** Temperate bacteriophages can integrate their genomes into the bacterial host chromosome and exist as prophages whose gene products play key roles in bacterial fitness and interactions with eukaryotic host organisms. Most bacterial chromosomes contain “cryptic” prophages that have lost genes required for production of phage progeny but retain genes of unknown function that may be important for regulating bacterial host physiology. This study provides such an example, where a cryptic-prophage-encoded product can perform multiple roles in the bacterial host and influence processes, including metabolism, cell division, and susceptibility to phage infection. Further functional characterization of cryptic-prophage-encoded functions will shed new light on host-phage interactions and their cellular physiological implications.

## Abstract (precis)

Temperate bacteriophages can integrate their genomes into the bacterial host chromosome and exist as prophages whose gene products play key roles in bacterial fitness and interactions with eukaryotic host organisms. Most bacterial chromosomes contain “cryptic” prophages that have lost genes required for production of phage progeny but retain genes of unknown function that may be important for regulating bacterial host physiology. This study provides such an example, where a cryptic-prophage-encoded product can perform multiple roles in the bacterial host and influence processes, including metabolism, cell division, and susceptibility to phage infection. Further functional characterization of cryptic-prophage-encoded functions will shed new light on host-phage interactions and their cellular physiological implications.

## INTRODUCTION

Bacteriophages are abundant in the environment, with an estimated 10^31^ bacteriophage (phage) particles, and outnumber their bacterial hosts by a factor of 10 to 1 (1, 2). They are found in all ecosystems that harbor bacteria and play a vital role in driving bacterial evolution (3). Based on their life cycles, phages can be broadly classified as virulent or temperate. Virulent phages use a lytic life cycle, wherein they infect bacterial hosts, use the host cell’s resources to make more phage particles, and ultimately lyse the cell to release progeny virions into the environment. Temperate phages can grow using a lytic life cycle or, alternatively, can undergo lysogeny, integrating their genomes at a specific attachment site in the host chromosome and remaining stably associated with the host. A bacterium with an integrated phage genome (prophage) is called a lysogen. Changes in host metabolic conditions or external environmental triggers can induce the prophage, which then excises out of the host chromosome and resumes a lytic life cycle (4, 5).

Nearly half of all sequenced bacterial genomes have been found to contain at least one prophage, with many genomes containing multiple prophages (6). Lysogeny comes at a cost to the bacterial host due to the extra burden of replication of prophage DNA and the threat of lysogen induction, which is lethal to the host cell. On the other hand, there are many well-documented examples of lysogenic conversion, where prophage-encoded products confer new and advantageous characteristics on the host (7, 8). Many prophages carry virulence genes that contribute to the pathogenicity of a bacterial host, e.g., phage-encoded Shiga toxin in Escherichia coli O157 strains (9), phage-encoded diphtheria toxin in Corynebacterium diphtheriae (10), and neurotoxin in Clostridium botulinum (11). Prophage-encoded toxins, host cell invasion factors, and serum resistance proteins promote various aspects of the infection processes carried out by bacterial pathogens (7). Another well-documented benefit of prophages is superinfection immunity. In a mixed population of lysogens and other bacteria, if a prophage becomes induced and lyses a host cell, the active phage particles released infect and lyse only the nonlysogens, while the lysogens are protected by the prophage-encoded immunity functions (5). Less well characterized at a mechanistic level are examples of prophage genes that increase the host’s ability to grow under different environmental or stress conditions (12–14).

Growing evidence suggests that in many genomes, most of the resident prophages are cryptic (defective), having suffered mutations that leave them unable to excise from the host chromosome, lyse host cells, or produce infectious phage particles (15–18). A recent study identified and characterized orthologous prophages that were integrated into an ancestral host genome and subsequently passed down vertically with the host chromosome in E. coli and *Salmonella* (16). Most of these prophages showed evidence of loss of large portions of the original prophage genome, but the remaining genes were under purifying selection (16). These results suggest that certain prophage genes are selected for during host evolution because they encode products that are advantageous to the host under some condition. The cryptic prophages of E. coli K-12 have been associated with several host phenotypes, including biofilm formation, stress sensitivity, and antibiotic resistance (19). To understand the molecular basis of cryptic-prophage-associated phenotypes, functional characterization of prophage genes is essential.

In E. coli K-12, the cryptic prophage Qin carries an operon encoding a small protein, DicB, and a small RNA (sRNA), DicF, that both function as cell division inhibitors (20–25). The sRNA DicF represses *ftsZ* translation by directly base pairing with the *ftsZ* mRNA near the Shine-Dalgarno sequence (24, 25). DicF also regulates other mRNAs that encode a variety of regulatory and metabolic functions (25). The 62-amino-acid protein DicB inhibits cell division by directly interacting with MinC and recruiting it to the septum via interactions with the septal protein ZipA, where MinC stimulates depolymerization of the Z ring, resulting in cell filamentation (23, 26–28). The region immediately upstream of the *dicBF* operon includes *dicA* and *dicC* and is similar in sequence and structural arrangement to the lambdoid phage immunity locus. DicA is analogous to the P22 phage C2 repressor and DicC to the P22 Cro repressor (29). DicA represses the *dicBF* operon promoter (which is similar to the λ phage P_L_ promoter), and the natural conditions leading to induction of the operon are unknown (29). DicB and DicF are conserved in many strains of E. coli, and, interestingly, many pathogenic strains of E. coli possess multiple cryptic prophages including *dicBF* operons (25, 30, 31).

In this study, we identified a role for the E. coli *dicBF* operon in resistance to bacteriophage infection. Short-term expression of the *dicBF* operon promotes E. coli resistance to λ phage infection. The resistance phenotype is primarily attributable to DicB. DicB does not affect λ phage adsorption to host cells. Instead, our results suggest that DicB inhibits injection of λ DNA into the cytoplasm through the inner membrane proteins ManYZ, which are components of the mannose phosphotransferase system. Consistent with an effect of DicB on ManYZ activity, we found that growth of *dicB*-expressing cells on minimal medium with mannose as the sole carbon source was strongly inhibited. Our results suggest that products encoded by the *dicBF* operon, found in cryptic prophages in many E. coli and *Shigella* strains, can impact bacterial physiology, including by altering the cells’ susceptibility to bacteriophage infection. We postulate that this may be a common reason why certain cryptic-prophage genes are retained in host chromosomes.

## RESULTS

### Transient induction of the *dicBF* operon protects against λ phage infection.

**FIG 1:**
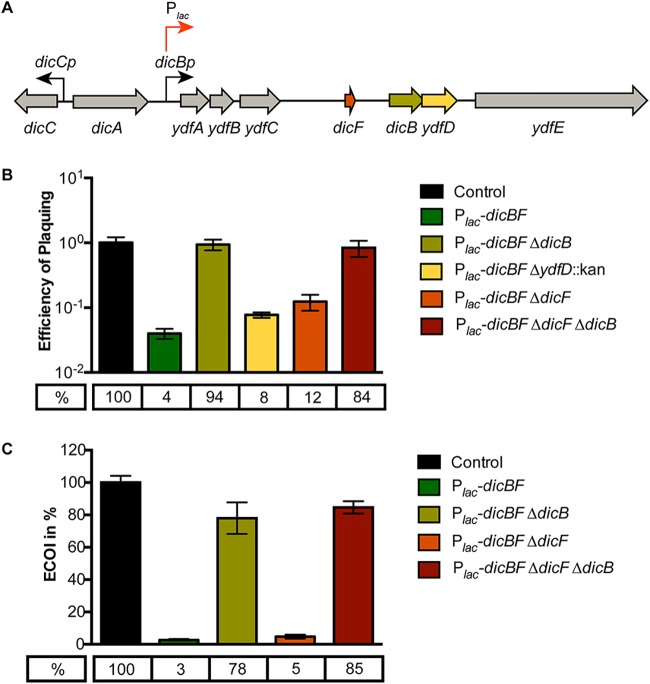
Transient induction of the *dicBF* operon protects against λ*vir* infection. (A) *dicBF* locus on the Qin prophage of E. coli K-12. The red arrow indicates where P*_lac_* is inserted on the chromosome, replacing the native *dicBp* promoter. (B) EOP is defined as the λ*vir* titer on the test strain divided by the λ*vir* titer on the control strain. The strains used in the experiment were DJ480 (control), DB240 (P*_lac_-dicBF*), PR165 (P*_lac_-dicBF* Δ*dicB*), DB247 (P*_lac_-dicBF* Δ*dicF*), PR164 (P*_lac_-dicBF* Δ*ydfD*::kan), and DB248 (P*_lac_-dicBF* Δ*dicF* Δ*dicB*). All the strains were grown to the same state of growth with the *dicBF* operon induced with 0.5 mM IPTG for 60 min. The inducer was washed off, and the strains were resuspended in TM buffer, infected with λ*vir*, and plated to calculate the titer. The error bars were calculated as standard deviations of values from three biological replicates. (C) ECOI is calculated as follows: (number of infectious centers per milliliter from the test strain) × 100/(number of infectious centers per milliliter from the control strain). The strains used in the experiment were DJ480 (control), DB240 (P*_lac_-dicBF*), DB243 (P*_lac_-dicBF* Δ*dicB*), DB247 (P*_lac_-dicBF* Δ*dicF*), and DB248 (P*_lac_-dicBF* Δ*dicF* Δ*dicB*). The cells were grown with induction of the *dicBF* operon with 0.5 mM IPTG and infected with λ*vir* at an MOI of 0.1. The unadsorbed phages were removed, and the phage-host complex was added to phage-sensitive cells (DJ480) and plated onto LB agar for plaque counting (infectious centers). The error bars were calculated as standard deviations of values from three biological replicates.

The region of the Qin prophage containing the *dicBF* operon (Fig. 1A) resembles the immunity regions of P22 and other lambdoid phages (29). While functions have not been identified for most of the products of the *dicBF* operon, DicB (a small protein) and DicF (a small RNA) have been shown to inhibit cell division (20, 22–25). We showed previously that DicF posttranscriptionally regulates a variety of genes involved in cell division, growth, and metabolism (25). Given their positions in the immunity region of the prophage genome and the fact that the characterized gene products impact cell physiology, we hypothesized that products of the *dicBF* operon could cause changes in the host cell that promote resistance to phage infection. We tested this by comparing phage infections of control and *dicBF*-expressing cells. Though some recent studies have found higher expression of the sRNA DicF under microaerobic or anaerobic conditions (31, 32), we have not yet found a condition that stimulates production of detectable levels of DicF or the polycistronic *dicBF* mRNA from the chromosomal locus in our strain. Thus, to test our hypothesis, we used an inducible expression system we described previously (25), where the *dicBF* operon promoter was replaced with a P*_lac_* promoter at the native locus. In addition to the P*_lac_-dicBF* strain, we used strains with deletions of different genes in the operon (Fig. 1B).

We measured phage infection of the strains by efficiency-of-plaquing (EOP) assays, initially using phage λ. In this assay, the titer of the phage is determined in all bacterial strains, and the titer (in PFU per milliliter) is calculated for each strain. The EOP is defined as the phage titer on the test strain divided by the phage titer on the control strain. The control strain lacked the P*_lac_* promoter. Strains with the P*_lac_* promoter driving *dicBF* expression were exposed to isopropyl-β-d-thiogalactopyranoside (IPTG) for 60 min. Then, the strains were infected with λ*vir* (a λ mutant that grows only via the lytic (and not the lysogenic) cycle during infection of host cells) and plated to determine its titer as described in Materials and Methods. The EOP for the P*_lac_-dicBF* strain was 0.04 (Fig. 1B), meaning that the rate of infection of the *dicBF*-expressing strain was only 4% relative to the control strain. This result suggested that transient expression of the *dicBF* operon conferred resistance to infection by λ*vir*. To further characterize the basis for this phenotype, we deleted *dicF*, *dicB*, and *ydfD*, singly and in combination, because previous studies identified growth or cell division phenotypes associated with these genes (23, 25, 33). The phenotypes of deletion mutants demonstrated that *dicB* played the most prominent role in the resistance phenotype (Fig. 1B). Deletion of *dicB* alone or *dicB* in combination with *dicF* restored the EOP of λ*vir* to nearly that of the control. In contrast, deletion of *dicF* or *ydfD* alone had a minimal effect on the resistance phenotype (Fig. 1B).

We observed previously that insertion of the P*_lac_* promoter upstream of the *dicBF* locus leads to low-level expression of the operon even in the absence of the inducer (25). We checked the EOP of λ*vir* on the P*_lac_-dicBF* strain with and without induction. In the absence of IPTG, the EOP was 58% compared with 4% in the presence of inducer (see Fig. S1 in the supplemental material). This result is consistent with leaky expression from the P*_lac_* promoter. We also carried out infections using the same host strains with wild-type λ phage and saw similar results for EOP on control, *dicBF*-expressing, and deletion mutant strains (see Fig. S2 in the supplemental material).

Because previous studies showed that ectopic expression of the *dicBF* operon impairs growth of the host strain, we reasoned that poor growth of test strains could influence the results of EOP assays. To more accurately assess the outcome of a phage infection on cells expressing the *dicBF* operon, we conducted center of infection (COI) assays. For this assay, strains (Fig. 1C) were induced with 0.5 mM IPTG, and λ*vir* infection was carried out at a multiplicity of infection (MOI) of 0.1. After adsorption of the phage to the test strains, the unadsorbed phages were removed by washing, and the infected test cells were diluted and mixed with the phage-sensitive control strain. Productive infections of the test strain were detected as plaques (centers of infection) on the phage-sensitive control strain. The efficiency of λ*vir* forming centers of infection (ECOI) was calculated as follows: (number of centers of infection per milliliter from the test strain) × 100/(number of centers of infection per milliliter from the control strain). The ECOI for λ*vir* on P*_lac_-dicBF* cells was 3% (Fig. 1C). This result is similar to the results of EOP assays (Fig. 1B), suggesting that the growth characteristics of the P*_lac_-dicBF* test strain did not impact the experimental outcome. Deletion of *dicB*, alone or in combination with *dicF*, restored the ECOI to ∼80%. The Δ*dicF* strain gave an ECOI of 5% (Fig. 1C). These results are again consistent with our EOP experiments (Fig. 1B), implicating DicB as the major player in the phage resistance phenotype.

### The *dicBF* operon promotes resistance to λ, but not other phages.

**FIG 2:**
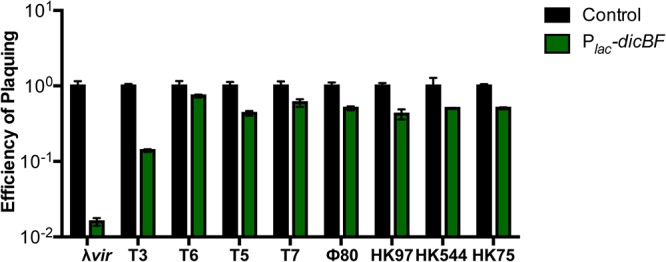
The *dicBF* operon confers resistance against λ phage but not other phages. For each of the nine phages, the titer (in PFU per milliliter) was calculated by infection of DJ480 (control) and DB240 (P*_lac_-dicBF*) cells. The cells were prepared for infection, and the EOP was calculated for each phage as described for Fig. 1B. The error bars were calculated as standard deviations of values from three biological replicates.

To determine if transient expression of *dicBF* conferred resistance to other phages, we conducted infection experiments using control and P*_lac_-dicBF* strains with nine different lytic and temperate phages (Fig. 2). In this experiment, the EOP of λ*vir* on the P*_lac_-dicBF* strain was 0.016, or 1.6%, compared to the control strain, which was the lowest of the nine phages tested (Fig. 2). Partial resistance was observed for the T3 phage, which had an EOP of 0.14 on P*_lac_-dicBF* cells. However, the EOPs for the remaining seven phages, including phages ϕ80 and HK97, which are closely related to λ, were similar to that for the control cells (Fig. 2). These results suggest that DicB does not provide a broad spectrum of resistance against bacteriophages.

### Effect of *dicBF* expression on λ phage growth.

The classical experiment to study the growth cycle of phages in bacteria is the one-step growth curve, as described by Ellis and Delbrück (34). They observed a latent period, when the numbers of phages recovered from infected cells remained low as new phage particles were being synthesized inside the host cell. After the latent period is the “burst,” when the numbers of infectious phage particles increase rapidly as the phage life cycle is completed and cells are lysed to release mature progeny. We conducted one-step growth curves for λ*vir* on control and *dicBF*-expressing strains, essentially as described above for ECOI experiments over a time course following infection. λ*vir* was added at an MOI of 0.1 to control and P*_lac_-dicBF* cells resuspended in TM buffer (10 mM Tris-HCl and 10 mM MgSO_4_). After phage adsorption, the cells were washed to remove unadsorbed phages, and the phage-host complexes were diluted in fresh medium with IPTG (see Materials and Methods). At each time point, the number of infectious phage particles in each culture was calculated by removing samples and plating for PFU on a phage-sensitive control strain.

**FIG 3:**
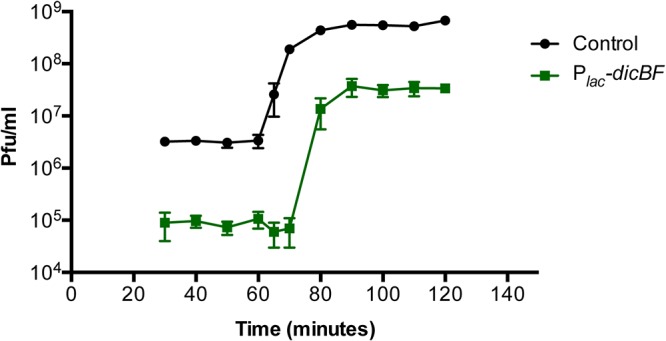
One-step growth curve of λ*vir* on control and P*_lac_-dicBF* cells. The cells were grown with induction of the *dicBF* operon, and infection was carried out at an MOI of 0.1, similar to the center of infection assay. After removing unadsorbed phages, the cells were diluted in LB medium (with IPTG to induce the *dicBF* operon) and incubated at 37°C for the entire duration of the growth curve. At each time point, starting at 30 min from the start of infection, samples were removed and added to the phage-sensitive strain (DJ480) and plated to count plaques. The burst size was calculated as follows: (phage titer at 100 min − initial titer at 30 min)/initial titer at 30 min. The latent period was calculated as the time at the midpoint of the exponential phase of the curve. The error bars were calculated as standard deviations of values from three biological replicates.

As expected based on previous results (Fig. 1B and C), the ECOI for λ*vir* on P*_lac_-dicBF* cells was reduced by almost 2 log units compared with the control strain at the early time points, and the reduced numbers of phages produced by P*_lac_-dicBF* cells persisted across the phage growth curve (Fig. 3). The latent period for P*_lac_-dicBF* cells (75 min) was ∼10 min longer than for control cells (65 min) (Fig. 3). The calculated burst sizes were 343 phages/P*_lac_-dicBF* cell compared to 169 phages/control cell. This increase in burst size in *dicBF*-expressing cells is likely due to filamentation of cells caused by DicB and DicF. It has been shown previously that filamenting cells produce more phages than normal-size cells (35, 36). Importantly, we observed that the ∼3% of phages that escaped DicB-mediated resistance followed a growth curve similar to that of phages growing on control cells. Collectively, these data led us to hypothesize that DicB affects an early step of the phage life cycle, like adsorption or DNA injection, since phages that escape this DicB effect complete a relatively normal life cycle.

### The *dicBF* operon does not affect phage adsorption to host cells.

To test if expression of the *dicBF* operon affects the first step of phage infection, we tested the ability of λ*vir* to adsorb to host cells expressing the operon. During the ECOI experiment, once phage infection was carried out with cells in TM buffer at an MOI of 0.1 and incubated at 37°C for 10 min, the phage-cell mixture was centrifuged and the supernatant containing the unadsorbed phages was removed. The titer of the supernatant was determined on phage-sensitive control cells by standard plaque assay (residual titer). The control titer was calculated using the same procedure described above, with phages added to TM buffer instead of bacterial cells in the first step of the experiment. The percent adsorption was calculated as follows: (control titer − residual titer) × 100/control titer.

The adsorption of λ*vir* to strains expressing the *dicBF* operon was the same as adsorption to the control strain (Table 1). Notably, while λ*vir* and HK97 both adsorb to the same outer membrane receptor, LamB (37, 38), the effects of *dicBF* expression on the EOP of these two phages are significantly different, with a reduced EOP only for λ*vir* (Fig. 2). These observations strongly suggest that DicB does not affect the phage life cycle at the step of adsorption to host cells.

**TABLE 1:** The *dicBF* operon does not affect phage adsorption to host cells^*a*^

Strain description^*b*^	% adsorption	±SE
Control	99	0.22
P*_lac_*-*dicBF*	99	0.25
P*_lac_*-*dicBF* Δ*dicB*	99	0.69
P*_lac_*-*dicBF* Δ*dicF*	99	0.23
P*_lac_*-*dicBF* Δ*dicF* Δ*dicB*	99	0.15

a The percent adsorption was calculated as follows: (control titer − residual titer) × 100/control titer. The standard error was calculated as the standard deviation of values from three biological replicates.

b The strains used in this experiment were DJ480 (control), DB240 (P*_lac_-dicBF*), DB243 (P*_lac_-dicBF* Δ*dicB*), DB247 (P*_lac_-dicBF* Δ*dicF*), and DB248 (P*_lac_-dicBF* Δ*dicF* Δ*dicB*). The cells were infected at an MOI of 0.1 with λ*vir*, which was allowed to adsorb for 10 min at 37°C. After adsorption, the samples were centrifuged, and the supernatant containing the unadsorbed phages was measured on phage-sensitive cells to quantify the titer (residual titer). The control titer was calculated by carrying out the assay with TM buffer without bacterial cells.

### Recombinant λ phages with the host range region of ϕ80 are not affected by DicB.

**FIG 4:**
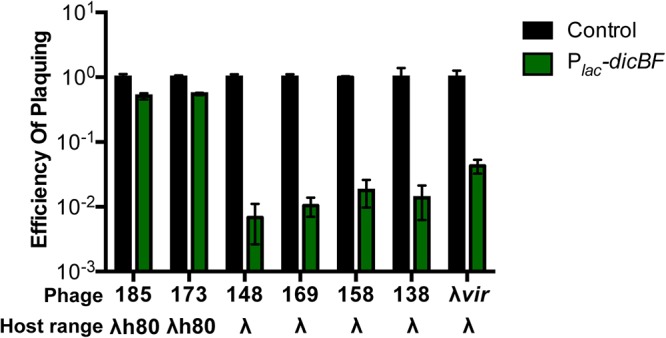
λ phage with the host range of ϕ80 is not affected by DicB. Recombinant λ phages with either the λ or ϕ80 host range were plaqued on DJ480 (control) or DB240 (P*_lac_-dicBF*) cells (see Table S1 for phage genotypes). The cells were prepared for infection, and the EOP was calculated for each phage as described for Fig. 1B. The error bars were calculated as standard deviations of values from three biological replicates.

The genomes of λ phage and ϕ80 (a lambdoid phage) have strikingly similar organizations, allowing easy construction of recombinant phages (39, 40). One prominent difference between λ and ϕ80 is their use of different outer and inner membrane receptors. λ uses LamB (outer membrane) and ManYZ (inner membrane) for adsorption and DNA injection, respectively (41–45), while ϕ80 uses FhuA (outer membrane) and the TonB complex (inner membrane) (39, 46, 47). The phage genes encoding determinants for utilization of host outer and inner membrane receptors are located in the host range region of lambdoid phage genomes. Our results so far suggest that the DicB-dependent phage resistance phenotype is not due to an effect on adsorption to the outer membrane receptor but might be mediated at another early step of infection, such as injection of the phage genome through the inner membrane receptor. To test this idea, we measured phenotypes of control and *dicBF*-expressing cells challenged with recombinant λ phage containing the host range region of ϕ80 (λh80). The λh80 phages carry most of the wild-type λ genome but have an altered host range region specifying use of the ϕ80 outer and inner membrane receptors. To confirm this, we tested the plaquing ability of λh80 phages on wild-type, Δ*fhuA*, Δ*tonB*, and Δ*manXYZ* E. coli strains. As expected, the λh80 phages, like ϕ80, did not plaque on Δ*fhuA* and Δ*tonB* strains but formed normal plaques on the Δ*manXYZ* strain (see Table S3 in the supplemental material). Next, we carried out EOP assays using λ*vir* and a panel of recombinant phages with the host range of λ or ϕ80 (see Table S1 in the supplemental material) on control and P*_lac_-dicBF* cells (Fig. 4). We hypothesized that if DicB mediates resistance to λ phage by impairing injection of phage DNA across the cytoplasmic membrane, then phages with the host range of λ would remain inhibited by DicB, whereas λh80 phages with altered inner membrane receptor specificity would not be impacted by DicB. The results of the EOP assays demonstrate that phages with the λ host range remained sensitive to DicB-mediated inhibition while λh80 phages had similar EOP on P*_lac_-dicBF* and control cells (Fig. 4). Together with our previous results, this observation suggests that DicB-mediated resistance acts at the level of the inner membrane receptor ManYZ used for λ phage DNA injection into the cytoplasm of E. coli. We note that the panel of phages that we tested in this experiment had other genetic differences, aside from the different host ranges (see Table S1). Only the host range was correlated with susceptibility to DicB-mediated resistance.

**FIG 5:**
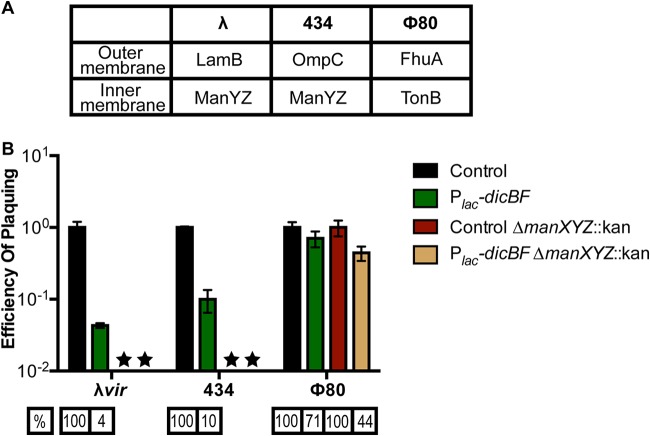
Phage 434 plaquing on ManYZ^+^ strains is inhibited by the *dicBF* operon. (A) Outer and inner membrane receptor specificities of phages λ*vir*, 434, and ϕ80. (B) An EOP assay was carried out by preparing cells and calculating the titers of phages on the different strains as described in the legend to Fig. 1B. The strains used in the experiment were DJ624 (control), DB240 (P*_lac_-dicBF*), PR187 (control Δ*manXYZ*::kan), and PR191 (P*_lac_-dicBF* Δ*manXYZ*::kan). The EOP of phages for each strain was calculated with respect to the control strain in the same background. The error bars were calculated as standard deviations of values from three biological replicates. The stars represent strains for which plaques could not be counted accurately, since the plaques were smaller than pinpoints. This has been observed before for λ plaques on Δ*manXYZ* mutant strains (44).

Phage 434 (44) is another phage that uses ManYZ for injection of DNA through the cytoplasmic membrane (Fig. 5A). Previous studies have shown that *manXYZ* deletion mutants (also known as *pel* mutants) were resistant to infection by λ and phage 434, but not ϕ80 (44). To further test our hypothesis that DicB inhibits phage infection at the level of DNA entry through ManYZ, we tested the abilities of λ*vir*, phage 434, and ϕ80 to infect control and *dicBF*-expressing cells in *manXYZ^+^* and Δ*manXYZ* backgrounds. We verified that λ, phage 434, and ϕ80 plaqued as expected on the wild-type strain and strains with mutations in specific receptors (see Table S4 in the supplemental material). As shown in Fig. 2, ϕ80 plaquing efficiency is not impacted by expression of the *dicBF* operon in a *manXYZ*^+^ background. The Δ*manXYZ* mutant host also supported wild-type EOP for ϕ80 plaquing regardless of whether *dicBF* was expressed (Fig. 5B). For λ*vir*, the EOP on P*_lac_-dicBF* cells was 4% relative to control in *manXYZ^+^* cells, whereas the *ΔmanXYZ* host did not support λ*vir* growth (Fig. 5B). The pattern of growth for phage 434 was very similar to that of λ*vir*, with a reduced EOP of ∼10% on P*_lac_-dicBF* cells in the *manXYZ^+^* host and no plaques on the Δ*manXYZ* host (Fig. 5B). The results of this experiment are consistent with the hypothesis that DicB inhibits the use of the mannose phosphotransferase system (PTS) proteins ManYZ as an inner membrane receptor for productive phage infection.

### Growth of *dicBF*-expressing cells is inhibited on plates with mannose as the C source.

Our previous results pointed to the small protein DicB inhibiting the activity of the mannose transporter ManYZ proteins with regard to DNA uptake during phage infection. To test whether DicB inhibits the functions of these proteins more broadly, we checked the growth of *dicBF*-expressing cells on mannose as the sole C source. For this experiment, we used control, P*_lac_-dicBF*, and P*_lac_-dicBF* Δ*dicB* strains. The strains were streaked on M63 minimal plates with different sugars with or without 0.025 mM IPTG (to induce *dicBF* expression) and incubated for 44 h at 37°C. Growth was scored using the parent strain’s growth as a reference (see the legends to Table 2 and to Fig. S3 in the supplemental material and examples of growth phenotypes in Fig. S3). In the absence of inducer, all the strains had nearly normal growth on the different sugars used. When *dicBF* expression was induced using 0.025 mM IPTG, we observed growth inhibition of P*_lac_-dicBF* cells on mannose and glucosamine, but not on glucose (see Fig. S3), fructose, or *N*-acetylglucosamine (Table 2). On glucosamine plates, we observed apparent suppressors of the P*_lac_-dicBF* strain on the plate with IPTG, which resulted in uneven growth of dense and light streaks on that plate. Comparing the single-colony sizes on the glucose-plus-IPTG and glucosamine-plus-IPTG plates demonstrates clearly that growth of the P*_lac_-dicBF* strain is inhibited on glucosamine (see Fig. S3). Deletion of *dicB* relieved the growth inhibition on mannose and glucosamine (Table 2; see Fig. S3). Both mannose and glucosamine sugars are transported via the ManXYZ transporter in E. coli (48–50). These results demonstrate that DicB affects growth specifically on substrates of ManYZ. Growth on sugars that are transported by other PTS proteins was unaffected. These data suggest that DicB impacts at least two different functions of ManYZ: uptake of phage DNA during infection and transport of sugar substrates.

**TABLE 2:** Growth of *dicBF*-expressing cells is inhibited on plates with mannose as the C source

C source	Growth^*a*^					
	Control		P*_lac_-dicBF*		P*_lac_-dicBF* Δ*dicB*	
	Uninduced	Induced	Uninduced^*b*^	Induced	Uninduced^*b*^	Induced
Mannose	+++	+++	++	+	+++	+++
Glucose	+++	+++	+++	+++	+++	+++
Glucosamine	+++	+++	++	+	+++	+++
Fructose	+++	+++	+++	+++	+++	+++
*N*-Acetylglucosamine	+++	+++	+++	+++	+++	+++

a The strains were streaked on M63 minimal medium with 0.2% sugars as the C source with or without 0.025 mM IPTG and incubated for 44 h at 37°C. The strains used were DJ624 (control), DB240 (P*_lac_-dicBF*), and PR165 (P*_lac_-dicBF* Δ*dicB*). +++ indicates growth of the control strain on the respective sugars; ++ and + indicate decremental growth based on the sizes of the single colonies on the plate compared to the control strain on that sugar (for examples of growth phenotypes corresponding to each score, see Fig. S3).

b The P*_lac_* promoter is leaky, and we suspect low-level expression of the *dicBF* operon even at 0 mM IPTG.

### MinC mutants that do not interact with DicB lose the phage resistance and sugar phenotypes.

The only characterized activity of DicB is inhibition of cell division (20, 51). The mechanism by which DicB impacts cell division requires a protein-protein interaction with MinC, one of the proteins involved in controlling septal ring placement in E. coli. MinC is an inhibitor of FtsZ polymerization, and normally MinC concentrations are highest at cell poles, so that septum formation is inhibited at polar sites and directed instead to midcell (52). Previous work demonstrated that DicB interacts with MinC and brings it to midcell via an interaction with ZipA, a septal protein (28). DicB-mediated localization of MinC to the cell center inhibits FtsZ polymerization and promotes filamentation (27, 28). To determine if the DicB-MinC interaction is necessary for the DicB-dependent phenotypes we found in this study, we constructed mutant strains that produce MinC proteins that are defective for interaction with DicB. We used two different MinC mutants: MinC R172A, which interacts weakly with DicB, and MinC E156A, which does not interact with DicB (53). In strains expressing these *minC* alleles, DicB had a modest (MinC R172A) or no (MinC E156A) impact on cell division, consistent with their reduced binding to DicB. We used MinC E156A and R172A mutant hosts to test whether the DicB-mediated phage resistance or sugar growth phenotype required the DicB-MinC interaction.

**FIG 6:**
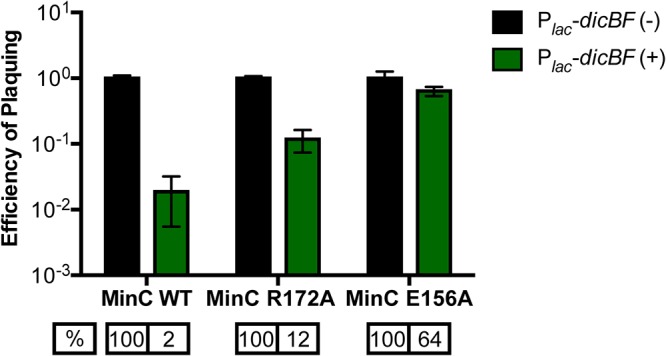
MinC mutants that do not interact with DicB lose the phage resistance effect. The cells were grown with induction of the *dicBF* operon with 0.5 mM IPTG and infected with λ*vir*, and the EOP was calculated as described in the legend to Fig. 1B. The EOP of λ*vir* for each strain was calculated with respect to the control strain in the same background. The strains used in the experiment were DJ624 (control), DB240 (P*_lac_-dicBF*), PR181 (control *minC* R172A), PR183 (P*_lac_-dicBF minC* R172A), PR180 (control *minC* E156A), and PR182 (P*_lac_-dicBF minC* E156A). The error bars were calculated as standard deviations of values from three biological replicates. WT, wild type.

As observed previously, in the wild-type *minC^+^* background, *dicBF*-expressing cells showed reduced EOP for λ*vir* compared to control cells (Fig. 6). However, in the MinC R172A (reduced binding to DicB) strain, the resistance phenotype was diminished; *dicBF* expression in this host gave an EOP of 12% compared to the control strain. In the MinC E156A (abrogated binding to DicB) background, the EOP of λ*vir* on *dicBF*-expressing cells was very similar to that of the control strain (Fig. 6). These results suggested that the DicB-MinC interaction is required for DicB-mediated resistance to λ phage infection. The same strains were grown on M63 minimal plates with different sugars with or without 0.025 mM IPTG to induce the *dicBF* operon. As shown above, in the wild-type *minC^+^* background, expression of *dicBF* inhibited growth on plates with mannose and glucosamine, but not on plates with glucose (Table 3). In contrast, *dicBF* expression in *minC* mutant strains (E156A and R172A) did not inhibit growth on any of the sugars tested (Table 3). Collectively, these data indicate that the new DicB-associated phenotypes we have identified—phage resistance and inhibition of growth on sugars that are transported by ManYZ—require the previously defined molecular mechanism of DicB interaction with the host protein MinC.

**TABLE 3:** MinC mutants that do not interact with DicB regain the ability to grow on mannose and glucosamine

Strain description	Growth^*a*^					
	Glucose		Mannose		Glucosamine	
	Uninduced	Induced	Uninduced	Induced	Uninduced	Induced
Control	+++	+++	+++	+++	+++	+++
P*_lac_-dicBF*	+++	+++	++	+	++	+
Control *minC* R172A	+++	+++	+++	+++	+++	+++
P*_lac_-dicBF minC* R172A	+++	+++	+++	+++	+++	+++
Control *minC* E156A	+++	+++	+++	+++	+++	+++
P*_lac_-dicBF minC* E156A	+++	+++	+++	+++	+++	+++

a The strains were streaked on M63 minimal medium plates with 0.2% sugars and 0.025 mM IPTG to induce the *dicBF* operon. The plates were incubated for 44 h at 37°C. The strains used in the experiment were DJ624 (control), DB240 (P*_lac_-dicBF*), PR181 (control *minC* R172A), PR183 (P*_lac_-dicBF minC* R172A), PR180 (control *minC* E156A), and PR182 (P*_lac_-dicBF minC* E156A). Growth on the plates was scored as described for Table 2. The P*_lac_* promoter is leaky, and we suspect low-level expression of the *dicBF* operon even at 0 mM IPTG.

## DISCUSSION

**FIG 7:**
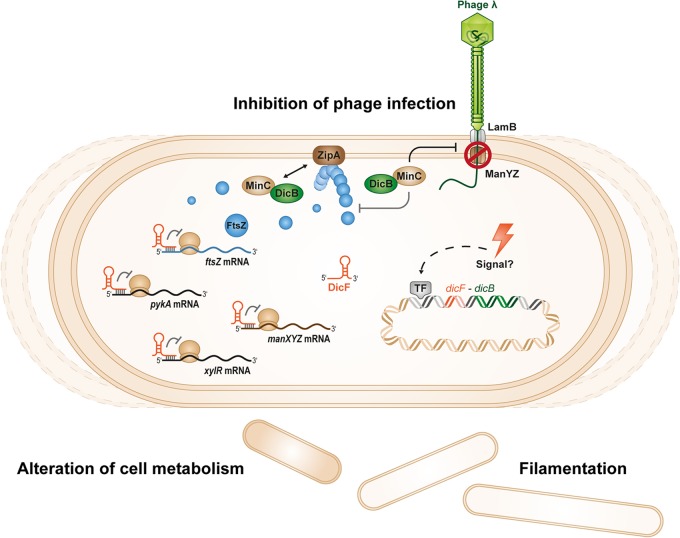
Working model for regulation of host cell physiology by DicF and DicB. The small protein DicB and the sRNA DicF of the *dicBF* operon are encoded on the Qin cryptic prophage of E. coli K-12. The physiological conditions that induce expression of the operon are not yet known. However, when ectopically expressed, DicB and DicF regulate multiple processes in the cell. DicF inhibits cell division and metabolism genes posttranscriptionally, including *manXYZ*, encoding the mannose PTS. DicB interacts with MinC, which is a negative regulator of FtsZ polymerization. The DicB-MinC complex interacts with the septal ring component ZipA and causes depolymerization of FtsZ at the septum, which leads to filamentation of cells. In this study, we showed that DicB confers on the host cell resistance to phages that use the inner membrane proteins ManYZ to inject their DNA into the cell. DicB also affects growth on mannose and glucosamine sugars, which are transported into the cell via ManXYZ. Interaction with MinC is necessary for DicB to promote the phage resistance and sugar phenotypes.

The existence of cryptic or defective prophages on bacterial chromosomes was discovered long ago (4), but their potential beneficial functions for host cells are still coming to light. In part, this is because we do not know the functions of the majority of genes carried on these prophages. In this study, we have identified a new functional role for the cryptic-prophage-encoded protein DicB in E. coli K-12. We showed that induction of the *dicBF* operon makes cells resistant to infection by phages that use the ManYZ PTS proteins as inner membrane receptors for DNA injection (Fig. 1 and 5). DicB, a 62-amino-acid protein encoded by the *dicBF* operon, plays the primary role in conferring this phage resistance phenotype (Fig. 1). Our results are consistent with the model that DicB inhibits phage DNA injection through the mannose transporter proteins ManYZ (Fig. 4, 5, and 7). The DicB effect on ManYZ also inhibits ManYZ-dependent transport of sugar substrates (Table 2), suggesting that DicB affects the general structure or function of these transport proteins. Previous work demonstrated that DicB inhibits cell division by interacting with and affecting the localization and activity of the cell division proteins MinC and FtsZ (23, 26–28). In this study, we found that the DicB-associated phage resistance and sugar utilization phenotypes are dependent on DicB-MinC interactions (Fig. 6 and Table 3).

Prior to this work, the only known function of DicB was inhibition of cell division. DicB directly interacts with MinC of the Min system, which consists of the proteins MinC, MinD, and MinE, which play roles in the spatial positioning of the FtsZ ring at midcell for cell division. MinC is the only component of the Min system required for DicB-dependent cell division inhibition (23). MinC is a negative regulator of FtsZ polymerization, and in E. coli, MinC oscillates between the two cell poles (driven by MinD and MinE) in order to inhibit Z ring assembly at the poles (54, 55). However, when DicB is expressed, a DicB-MinC complex is formed which interacts with the septal ring component ZipA and stimulates Z ring depolymerization at midcell, leading to cell filamentation (28). Both activities of DicB, cell division inhibition and the ManYZ inhibition phenotypes reported in this study, involve interaction with MinC. These results imply a previously unsuspected link between the Min system or other components of the cell division machinery and the mannose PTS. A few studies have examined localization of various PTS proteins. The general PTS proteins, EI and HPr, were found to localize primarily to cell poles (56, 57). Localization of EII sugar permeases has been less studied, but there is recent evidence that these proteins cluster together around the cell membrane (58). It will be interesting in future work to explore the subcellular localization of ManYZ and to examine if or how it is impacted by MinC and DicB.

It is clear that active prophages can protect their hosts from superinfection by other phages (59, 60), a phenotype called superinfection exclusion. One mechanism of superinfection exclusion is mediated by prophage-encoded proteins that block entry of a superinfecting phage’s DNA by mechanisms that are not well defined but may involve modifying the activity or function of inner membrane receptors (38, 59, 61–64). Our results suggest that in at least some cases defective prophages can play similar roles in protecting their hosts from phage infection. It has been speculated that another beneficial role of defective prophages could be encoding functions that are important for host cell adaptation to stress conditions. In a study by Wang et al. (19), defective prophages of E. coli K-12 were shown to increase resistance to environmental stresses, like oxidative stress and osmotic stress, and to certain antibiotics, like quinolones and beta-lactams. The study reported that Δ*qin* derivatives of the parent strain were more sensitive to beta-lactam antibiotics and that Δ*dicB* strains showed greater sensitivity to azlocillin and nalidixic acid (19). We constructed Δ*qin* and Δ*dicB* strains and examined their sensitivities to nalidixic acid and ampicillin, and we found no differences in sensitivity between parent strains and mutants (data not shown). It is possible that phenotypes vary with strain background—our strains are MG1655 derivatives, and Wang et al. used BW25113 (19).

Identifying the signals or conditions that induce prophage genes will be key to understanding their physiological roles in host cells. We have exposed our strains to various conditions that are known to induce prophage gene expression, including DNA damage, starvation, and exposure to antibiotics, and have not yet identified conditions that substantially induce transcription from the native *dicBF* promoter (data not shown). Another study (32) reported that E. coli K-12 MG1655 cells undergo DicF-dependent filamentation under anaerobic conditions (growth in large-volume anaerobic fermentors). The authors of the study suggest that the stability of DicF is differentially regulated so that it is more stable under anaerobic growth conditions and degrades faster under aerobic conditions. A very recent study found that four DicF orthologs encoded by different prophages in E. coli O157:H7 are produced under microaerobic growth conditions (31). These DicF sRNAs promote low-oxygen-responsive virulence gene expression via base pairing-mediated regulation of a key virulence transcription factor. These studies suggest that in at least some E. coli strain backgrounds, oxygen is an important signal for modulation of *dicBF* operon transcription or DicF mRNA stability. However, we have not observed any DicF- or DicB-mediated filamentation of MG1655 cells grown in small-volume LB liquid cultures in an anaerobic chamber (data not shown), so we speculate that additional signals or conditions might contribute to *dicBF* operon expression in our host strain background.

Previous studies from our laboratory characterized the mRNA target regulon of DicF (25). In addition to the previously discovered DicF target *ftsZ* mRNA, we found that DicF base pairs with and represses translation of *xylR*, *pykA*, and *manXYZ* mRNAs, encoding the xylose repressor, pyruvate kinase, and mannose PTS components, respectively (25, 65). Thus, the *dicBF* operon encodes a base pairing-dependent sRNA regulator (DicF) and a small protein (DicB) that act at different levels to inhibit the synthesis and activity of a PTS sugar transporter (ManXYZ) (Fig. 7). This is strikingly similar to the regulation of the glucose PTS (*ptsG*; enzyme IICB^Glc^) by the dual-function sRNA SgrS and the small protein it encodes, SgrT. SgrS base pairs with and represses translation of *ptsG* mRNA (66, 67), while SgrT inhibits PtsG activity at a posttranslational level (68–70). Perhaps regulation of PTS enzyme synthesis and activity by sRNAs and small proteins is a common mechanism for posttranscriptional control of these systems. Future studies on the multitude of sRNAs and small proteins encoded on prophages and bacterial chromosomes promise to reveal more surprising connections between phages and their hosts.

## MATERIALS AND METHODS

### Strain construction and media.

All the strains and phages used in this study are summarized in Table S1 in the supplemental material, and the oligonucleotides (from Integrated DNA Technologies) are listed in Table S2. The strains used in the study are derivatives of E. coli K-12 strains MG1655 and BW25113. Chromosomal mutations were constructed using the λ red recombination method as described previously (71–73) or were moved into the required strain background using P1 transduction (74).

Construction of strain DB240, which has a P*_lac_* promoter inserted upstream of *ydfA* replacing the native *dicBF* operon promoter, was described previously (25). Oligonucleotides O-PR185 and O-PR186 were used to amplify the kanamycin resistance gene from pKD13, and the PCR product was recombined into the chromosome of DB240 using λ red functions produced by pSIM6 (73). The resulting Δ*dicB*::kan strain was called PR163. The kanamycin cassette was removed and replaced with an “FRT scar” using pCP20 (72) to create the Δ*dicB*::*scar* strain PR165. Similarly, a Δ*ydfD*::kan strain called PR164 was constructed by amplifying the kanamycin cassette of pKD13 using oligonucleotides O-PR189 and O-PR190 and recombining it into DB240.

A Δ*manXYZ*::kan deletion was moved into DJ624 and DB240 by P1 transduction from YS208 (75) to create PR187 and PR191, respectively. MinC mutants with single amino acid changes E156A and R172A (53) were constructed by first inserting a kan-*araC*-P_BAD_-*ccdB* PCR product into the *minC* gene in strain DJ624. Oligonucleotides O-PR209/O-PR210 (for E156A) and O-PR205/O-PR206 (for R172A) were used to amplify the kan-*ccdB* region of strain YS243, and the PCR product was recombined into DJ624(pSIM6) to generate strains PR178 and PR179, respectively. Oligonucleotides O-PR211 and O-PR212 (containing the E156A mutation) were used to amplify a segment of DNA from the control strain DJ480 to generate a PCR product with the desired mutations for *minC* E156A and recombined into PR178 pSIM6 to generate strain PR180. Oligonucleotides O-PR213 and O-PR214 (containing the R172A mutation) were used similarly to generate a PCR product with mutations for *minC* R172A and recombined into PR179 pSIM6 to create strain PR181. A P*_lac_* promoter replacing the promoter of the *dicBF* operon was introduced in PR180 and PR181 to generate strains PR182 and PR183, respectively.

E. coli K-12 strains were grown in LB medium at 37°C on a rotary shaker. All phage dilutions were made in TM buffer containing 10 mM Tris-HCl and 10 mM MgSO_4_, and phage infections were carried out using the same buffer. For phage infections, the top agar was made with equal parts LB agar and TM buffer, unless otherwise specified (76). Top agar was added to the infection mixture and plated on LB agar plates.

### Phage propagation.

New stocks of each phage were prepared as described by Rotman et al. (76). Plating cultures were prepared by growing DJ480 in tryptone broth (TB) medium with 5 mM MgSO_4_ (and 0.2% maltose exclusively for λ stock preparation) until late log phase, after which an equal amount of TM buffer was added and the mixture was vortexed vigorously. The titers of old phage stocks were determined by combining phage stocks with prepared plating cultures, mixing in top agar (made of equal parts TB agar and TM buffer), and plating onto TB agar plates that were subsequently incubated overnight at 37°C. The next day, a single individual plaque was punched out and incubated in TM buffer at room temperature for 1 to 2 h with occasional vortexing. Between 10 and 30 μl of the single-plaque eluate was mixed with 300 μl of DJ480 plating culture and incubated at 37°C for 15 min; 3 ml TB-TM top agar was added and plated onto TB plates for incubation at 37°C. After 3 to 7 h, when the lysis was confluent, the plate was overlaid with 5 ml TM buffer overnight at room temperature. The TM buffer containing phages was collected in the morning, and 4 ml fresh TM buffer was added to the plate and kept at room temperature. After 8 h, the remaining TM buffer containing phages was collected, and the combined eluate was centrifuged to pellet the agar and cells down. The supernatant was transferred into a fresh tube, 50 μl chloroform was added, and the fresh phage lysate was stored at 4°C.

### EOP assay.

The strains used in the EOP experiment were precultured from overnight cultures in LB and subcultured in LB medium to ensure all the strains were in the same state of growth when phage infection was carried out. After 1 h of subculturing (when the optical density at 600 nm [OD_600_] was ∼0.1 to 0.2), IPTG was added to a final concentration of 0.5 mM to induce the P*_lac_* promoter. After 1 h with IPTG induction, the cells were washed and resuspended in LB medium. The final OD_600_ was measured, and 1 ml of the culture was centrifuged and resuspended in 1 ml TM buffer; 100 μl of the phage dilution was added to 100 μl of bacteria from the previous step and incubated for 10 min at 37°C. After 10 min, 3 ml prewarmed LB top agar was added to the mixture and plated onto LB agar plates. The plates were incubated overnight at 37°C, and the plaques were counted. The EOP was calculated as the phage titer on the test strain (in PFU per milliliter) divided by the phage titer on the control strain (in PFU per milliliter) (77, 78).

### ECOI assay.

The strains were prepared for infection as described for the EOP assay. The only difference was in the last step of sample preparation, when the cells were resuspended in TM buffer with 0.5 mM IPTG to induce P*_lac_-dicBF* during phage infection. The procedure followed for the ECOI assay was based on that described by Moineau et al. (79). λ*vir* lysates were added to 500 μl of prepared strains at an MOI of 0.1 or less and incubated at 37°C for 10 min. The infection mixture was washed with TM buffer containing 0.5 mM IPTG to remove unadsorbed phages and resuspended in 500 μl of fresh buffer. The infected cells were diluted in TM-IPTG buffer, and 100 μl of each dilution of strains was added to 100 μl of DJ480 cells in TM buffer, LB-TM top agar was added to this mixture and plated onto LB agar plates. The plates were incubated overnight at 37°C, and the plaques arising from each individual infection were observed and counted. The ECOI was calculated as follows: (number of centers of infection per milliliter from the test strain) × 100/(number of centers of infection per milliliter from the control strain) (79).

### One-step growth curve.

The samples were prepared for infection as described for the ECOI assays. The one-step growth curve experiment was designed based on previously published methods (79). After resuspending the cells, λ*vir* was added at an MOI of 0.1 or less to 500 μl of cells and incubated for 10 min at 37°C. The infection mixture was washed to remove unadsorbed phages and resuspended in 500 μl of TM buffer with 0.5 mM IPTG. The strains were diluted 1:10,000 for DJ480 and 1:1,000 for DB240 (P*_lac_-dicBF*) to a final volume of 20 ml in LB with 10 mM MgSO_4_ and 0.5 mM IPTG in flasks and incubated in a 37°C water bath. Immediately, 100 μl was withdrawn from the flask and added to 100 μl phage-sensitive DJ480 cells in TM buffer (for lawn formation); prewarmed top agar was added to this mixture, and it was plated onto LB agar plates. The first time point was 30 min after the start of infection. The same procedure was repeated for each time point. The burst size was calculated as follows: (phage titer at 100 min − initial titer at 30 min)/initial titer at 30 min. The latent period was calculated as the midpoint of the exponential phase of the growth curve (79).

### Adsorption assay.

The procedure described above for ECOI assays was followed, and after the strains were infected with λ*vir* at an MOI of 0.1 and allowed to adsorb for 10 min at 37°C, the strains were centrifuged for 5 min at 13,000 rpm to pellet cells and adsorbed phages. One hundred microliters of the supernatant was removed, and dilutions were made in TM buffer; 10 μl of each dilution was added to 100 μl DJ480 (phage-sensitive) cells in TM buffer and incubated for 10 min at 37°C. This mixture was plated onto LB plates using top agar and incubated overnight at 37°C. The control titer was calculated using the same procedure described above with the same amount of phages required for an MOI of 0.1 added to 500 μl TM buffer (no bacteria). The percent adsorption was calculated as follows: (control titer − residual titer) × 100/control titer (77, 80).

### Growth on minimal medium plates with different sugars.

For growth assays, M63 minimal medium plates with sugars (glucose, fructose, mannose, *N*-acetylglucosamine, and glucosamine) at a final concentration of 0.2% were prepared with or without 0.025 mM IPTG. The strains were streaked on the plates and incubated for 44 h at 37°C. By visual inspection, the strains were scored for growth, with +++ denoting growth of the control strain on each sugar, ++ and + denoting decreased growth compared to the control strain on the respective sugar, and − indicating no growth.

## Supplementary Material

Supplemental file 1
