# Supplementary material for: Cryptic-Prophage-Encoded Small Protein DicB Protects Escherichia coli from Phage Infection by Inhibiting Inner Membrane Receptor Proteins
Source: J Bacteriol. 2019 Nov 5;201(23):e00475-19. doi: 10.1128/JB.00475-19 (PMC6832061; doi:10.1128/JB.00475-19)
Supplement: Supplemental file 1 [file JB.00475-19-s0001.pdf]

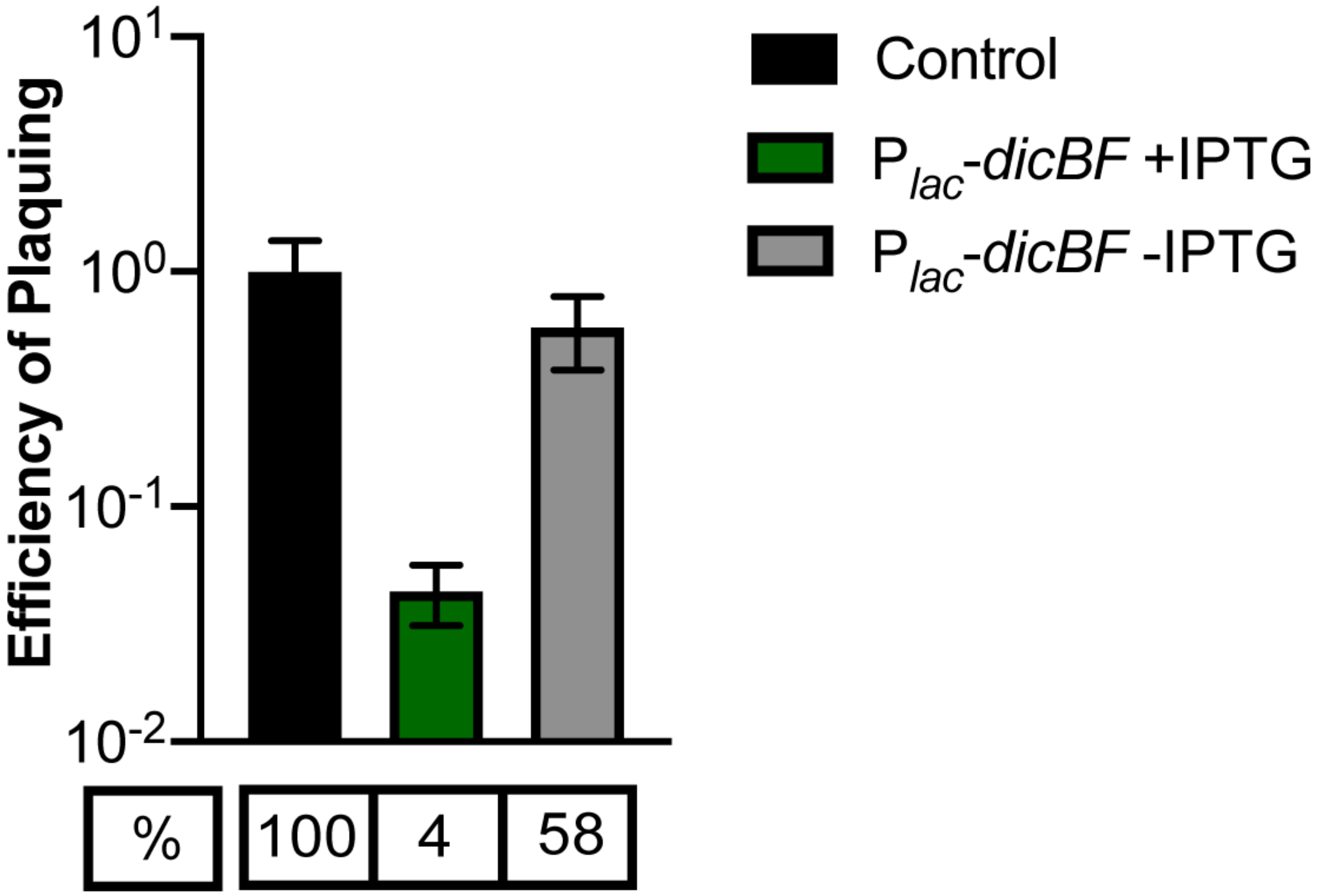

Fig. S1. Effect of the P_{lac} promoter on λ*vir* infection. EOP assay was carried out as described in Fig. 1B, without and with induction of the *dicBF* operon with 0.5mM IPTG. The strains used in this experiment are control (DJ624) and P_{lac}-*dicBF* (DB240).

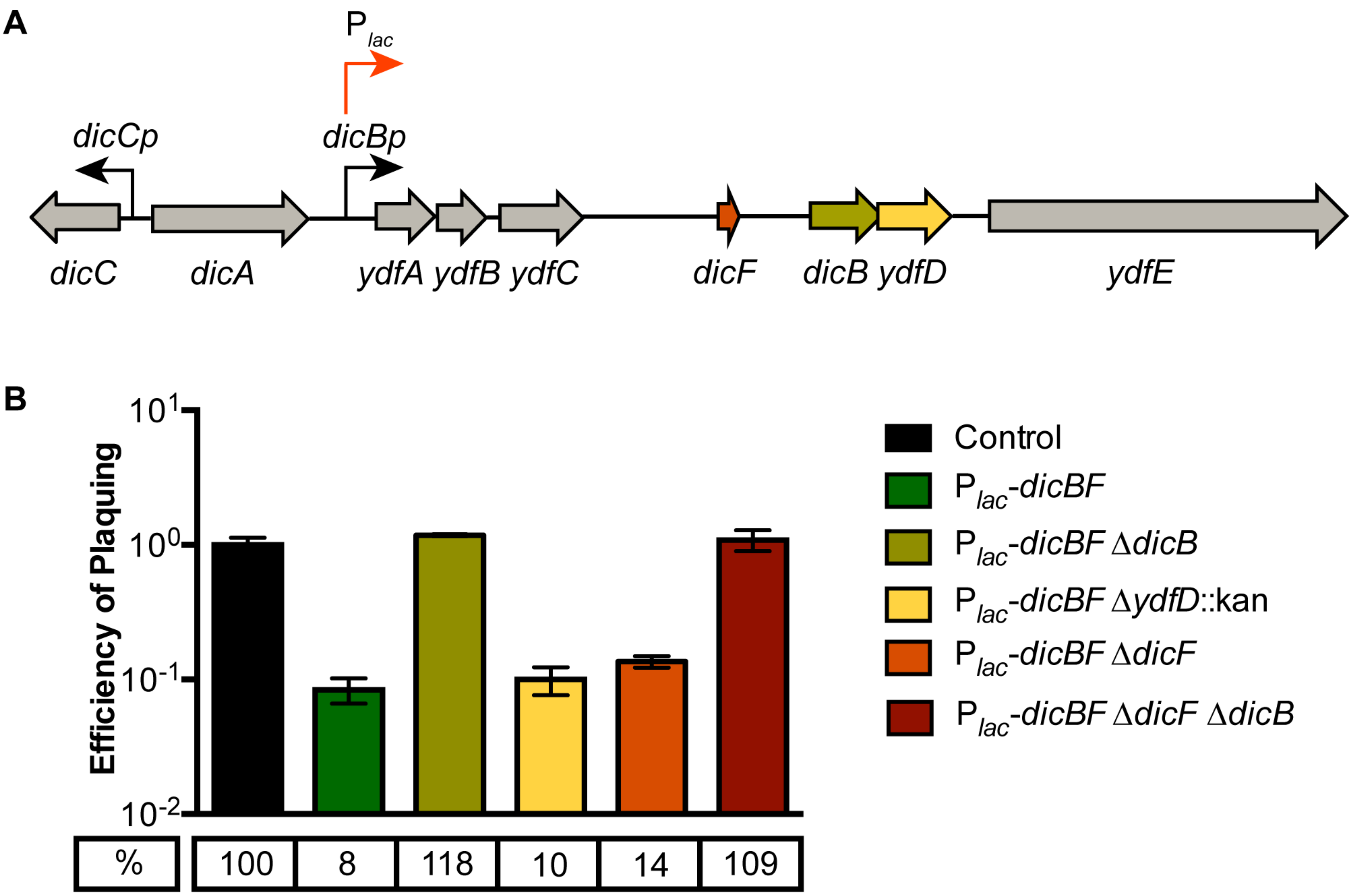

Fig. S2. Transient induction of the *dicBF* operon protects against wild-type λ infection. (A) The *dicBF* locus on Qin prophage of *E. coli* K-12. The red arrow indicates where P_{lac} is inserted on the chromosome, replacing the native *dicBp* promoter. (B) Strains used in this experiment are: Control (DJ480), P_{lac}-*dicBF* (DB240), P_{lac}-*dicBF* Δ*dicB* (PR165), P_{lac}-*dicBF* Δ*dicF* (DB247), P_{lac}-*dicBF* Δ*ydfD*::kan (PR164) and P_{lac}-*dicBF* Δ*dicF* Δ*dicB* (DB248). EOP was calculated as described for Fig. 1B.

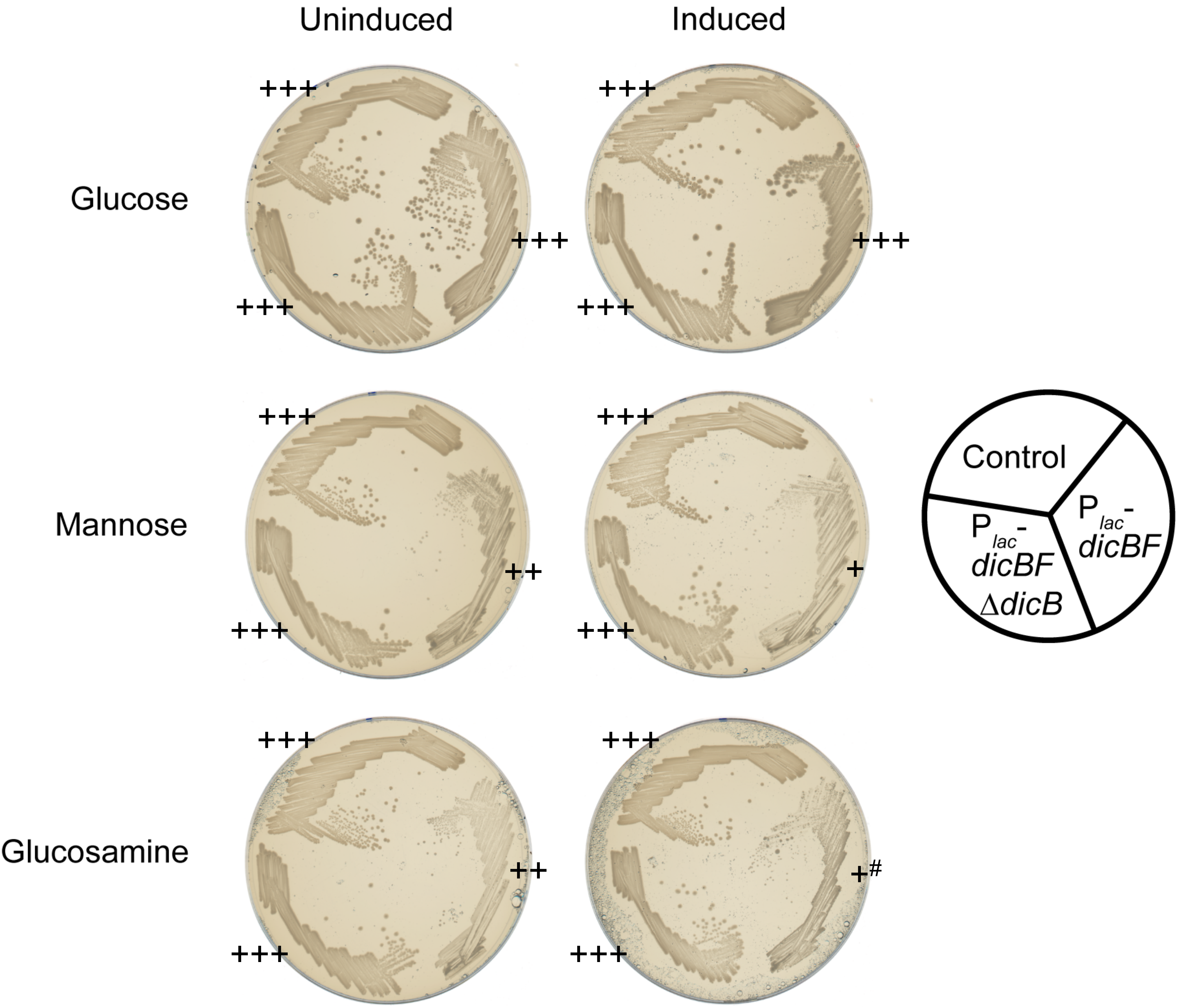

Fig. S3. Growth of *dicBF* expressing strains is inhibited on mannose and glucosamine plates. The strains were streaked on M63 minimal medium with 0.2% sugars as C source without or with 0.025mM IPTG and incubated for 44 hours at 37°C. The strains used are control (DJ624), P_{lac}-*dicBF* (DB240) and P_{lac}-*dicBF* Δ*dicB* (PR165). The P_{lac} promoter is leaky and we suspect low level expression of the *dicBF* operon even at 0mM IPTG. +++ indicates growth of the control strain on respective sugars, ++ and + indicates decremental growth based on the size of the single colonies on the plate compared to the control strain on that sugar. # indicates that P_{lac}-*dicBF* suppressor colonies arise on glucosamine plate with IPTG induction, which is explained in the Results section.

Strain name	Genotype	Source
DJ480	MG1655 Δ*lac* X74	D. Jin, NCI, Frederick, MD
DJ624	DJ480 *mal*::*lacI*^{q}	D. Jin
NM2000	DJ624 miniλ *tet*	N. Majdalani, NCI, Bethesda, MD
DB240	NM2000 *cat*-P_{lac}-*dicBF*	(1)
DB243	DB240 Δ*dicB*	(1)
DB247	DB240 Δ*dicF*	(1)
DB248	DB240 Δ*dicF*Δ*dicB*	(1)
PR163	DB240 Δ*dicB*::kan	This study
PR164	DB240 Δ*ydfD*::kan	This study
PR165	DB240 Δ*dicB*	This study
PR178	DJ624 Δ*minC*::kan-*araC*-P_{BAD}-*ccdB* (for E156A)	This study
PR179	DJ624 Δ*minC*::kan-*araC*-P_{BAD}-*ccdB* (for R172A)	This study
PR180	DJ624 *minC* E156A	This study
PR181	DJ624 *minC* R172A	This study
PR182	*cat*-P_{lac}-*dicBF minC* E156A	This study
PR183	cat-P_{lac}-*dicBF minC* R172A	This study
PR187	DJ624 Δ*manXYZ*::kan	This study
PR191	DB240 Δ*manXYZ*::kan	This study
YS208	DJ480 Δ*manXYZ*::kan	(3)
YS243	DJ480 Δ*sgrS*::kan-*araC*-P_{BAD}-*ccdB*	Lab collection
BW25113		J. Cronan, University of Illinois, Urbana, IL
BW25113 Δ*fhuA*::kan		(2)
BW25113 Δ*tonB*::kan		(2)
BW25113 Δ*lamB*::kan		(2)
BW25113 Δ*ompC*::kan		(2)
**Phage name**	**Genotype**	**Source**
λvir		J. Gardner, University of Illinois, Urbana, IL
λ wild-type		A. Kuzminov, University of Illinois, Urbana, IL
T3		A. Kuzminov
T6		A. Kuzminov
T5		A. Kuzminov
T7		A. Kuzminov
ϕ80		A. Kuzminov
HK97		A. Davidson, University of Toronto, Toronto, ON
HK544		A. Davidson
HK75		A. Davidson
185	λh80 Δ(*att int*) *i*21C (B274)	J. Gardner
148	λ *bio936* (*attR*$^{+}$) *imm*21^{ts}	J. Gardner
169	λ *gal8 bio936* (*attB*$^{+}$) *Jam imm*21^{ts}	J. Gardner
173	λh80 Δ9 [*att50-int50-red*] *imm*434 CI (G127)	J. Gardner
158	λ *bio16A* CI857	J. Gardner
138	λ *gal8* CI857	J. Gardner
434		S. Adhya, NCI, Bethesda, MD

Table S1. Strains and phages used in this study.

Literature cited

(1) Balasubramanian D, Ragunathan PT, Fei J, Vanderpool CK. 2016. A Prophage-Encoded Small RNA Controls Metabolism and Cell Division in *Escherichia coli*. mSystems 1:e00021-15.

(2) Baba T, Ara T, Hasegawa M, Takai Y, Okumura Y, Baba M, Datsenko KA, Tomita M, Wanner BL, Mori H. 2006. Construction of *Escherichia coli* K-12 in-frame, single-gene knockout mutants: the Keio collection. Mol Syst Biol 2:2006.0008.

(3) Sun Y, Vanderpool CK. 2013. Physiological Consequences of Multiple-Target Regulation by the Small RNA SgrS in *Escherichia coli*. J Bacteriol 195:4804–4815.

Oligonucleotides Name	Description	Sequence
O-PR185	Forward primer to delete *dicB*	CAGGCACTGCATCACAAAATTCATTGTTGAGGACGCGATA GTGTAGGCTGGAGCTGCTTC
O-PR186	Reverse primer to delete *dicB*	AAATGCTGAATTCATTGTGCACATCCTTTTGGCATCAGACA TTCCGGGGATCCGTCGACC
O-PR187	Forward test primer for *dicB* deletion	GCTGAGCGCACGCGGAACAG
O-PR188	Reverse test primer for *dicB* deletion	GTTCGGTTGCTGCAGTCATACACTCCTGCA
O-PR189	Forward primer to delete *ydfD*	TTTACGTCTGATGCCAAAAGGATGTGCACAATGAATTCAGG TGTAGGCTGGAGCTGCTTC
O-PR190	Reverse primer to delete *ydfD*	GCATATAAGAATGAAACCGGATATTTATTACGGAACTGTTA TTCCGGGGATCCGTCGACC
O-PR191	Forward test primer for *ydfD* deletion	GAAATTGGTGTCACTATCAGTAACCCAGTA
O-PR192	Reverse test primer for *ydfD* deletion	GTCAGGAATAGCGGCGCAAA
O-PR205	Forward primer to insert kan-*ccdB* in *minC* for R172A mutation	GATTGCCGATGGGAACATTCATGTCTATGGCATGATGCGC ATAGGAACTTCAAGATCCCCTTATTAGAAGAACTCG
O-PR206	Reverse primer to insert kan-*ccdB* in *minC* for R172A mutation	AAAATATTTGCGTTTCCCGGTCACCACTTGCCCCTGCCAGT TATATTCCCCAGAACATCAGGTTAATGGCG
O-PR207	Forward primer to check *minC*	CCGGTGCGTTCCGGTCAGCG
O-PR208	Reverse primer to check *minC*	TCAGCCAGTATTCACCTGCGATGGACACCA
O-PR209	Forward primer to insert kan-*ccdB* in *minC* for E156A mutation	CCACAATGTGATCTGATTGTTACAAGCCACGTTAGCGCTGA TAGGAACTTCAAGATCCCCTTATTAGAAGAACTCG
O-PR210	Reverse primer to insert kan-*ccdB* in *minC* for E156A mutation	ACCGCGCATCATGCCATAGACATGAATGTTCCCATCGGCA TTATATTCCCCAGAACATCAGGTTAATGGCG
O-PR211	Forward primer containing *minC* E156A mutation	CCACAATGTGATCTGATTGTTACAAGCCACGTTAGCGCTG GGGCCGCCTTGATTGCCGAT
O-PR212	Reverse primer containing *minC* E156A mutation	ACCGCGCATCATGCCATAGACATGAATGTTCCCATCGGCA ATCAAGGCGGCCCCAGCGCT
O-PR213	Forward primer containing *minC* R172A mutation	GATTGCCGATGGGAACATTCATGTCTATGGCATGATGCGC GGTGCGGCGCTGGCAGGGGC
O-PR214	Reverse primer containing *minC* R172A mutation	AAAATATTTGCGTTTCCCGGTCACCACTTGCCCCTGCCAG CGCCGCACCGCGCATCAT
O-PR214-2	Forward test primer for *manXYZ* deletion	TATCAGAGACGCCTCTGATTTGGCAAAGAT
O-PR215	Reverse test primer for *manXYZ* deletion	GATCGTCGATATTTCGAACGCGGATTAACA
O-PR222	Forward test primer for *minC* further upstream to O-PR207	TGCAAAGATGCGCAACTTAAAGCCG

Table S2. Oligonucleotides used to make strains in this study.

Strains	λ*vir*	ϕ80	173 (λh80)	185 (λh80)
DJ624	+	+	+	+
DJ624 Δ*manXYZ*::*kan*	*	+	+	+
BW25113	+	+	+	+
BW25113 Δ*fhuA*::*kan*	+	–	–	–
BW25113 Δ*tonB*::*kan*	+	–	–	–

Table S3. The phages used in Fig. 4 were titered on the strains listed to test their ability to form plaques. + denotes phage formed plaques, - denotes phage did not form plaques, and * denotes phage formed tiny plaques.

Strains	λvir (pfu/ml)	Phage 434 (pfu/ml)	ϕ80 (pfu/ml)
DJ624	$1.53*10^{10}$	$6.8*10^{8}$	$9.9*10^{9}$
DJ624 Δ*manXYZ*::*kan*	*	*	$3.2*10^{9}$
BW25113	$1.41*10^{10}$	$7.5*10^{8}$	$9.1*10^{9}$
BW25113 Δ*lamB*::*kan*	–	$8.7*10^{8}$	$1.26*10^{10}$
BW25113 Δ*ompC*::*kan*	$1.23*10^{10}$	–	$9.6*10^{9}$

Table S4. The phages used in Fig. 5B were titered on the different strains mentioned to test their ability to form plaques. - denotes phage did not form plaques and * denotes phage formed tiny plaques.
